# The use of functional and effective connectivity techniques to understand the developing brain

**DOI:** 10.1016/j.dcn.2015.01.011

**Published:** 2015-02-11

**Authors:** Diane Goldenberg, Adriana Galván

**Affiliations:** a Department of Psychology, University of California, Los Angeles, 1285 Franz Hall, Box 951563, Los Angeles, CA 90095, USA; b Brain Research Institute, University of California, Los Angeles, 695 Charles Young Dr. S., Los Angeles, CA 90095, USA

**Keywords:** Brain development, Functional connectivity, Effective connectivity, Resting state, Graph theory, Dynamic causal modeling

## Abstract

Developmental neuroscience, the study of the processes that shape and reshape the maturing brain, is a growing field still in its nascent stages. The developmental application of functional and effective connectivity techniques, which are tools that measure the interactions between elements of the brain, has revealed insight to the developing brain as a complex system. However, this insight is granted in discrete windows of consecutive time. The current review uses dynamic systems theory as a conceptual framework to understand how functional and effective connectivity tools may be used in conjunction to capture the dynamic process of change that occurs with development.

## Highlights

• Functional and effective connectivity have revealed the brain as a complex network.
• Developmental research is often limited in capturing the process of change.
• Dynamic systems theory offers a framework for developmental connectivity research.

## 1 Introduction

The brain is a complex and dynamic functional system, characterized by constant activity and change. Billions of neurons form intricate patterns that can flexibly integrate based on shared function, forming networks that are constrained by, but not limited to, direct structural connections of the brain (Vincent et al., 2007). Functional networks have the amassed capacity to support complex thought and action that any single element of the system would be unable to support alone. However, the topology of functional networks has been largely intangible until the relatively recent emergence of functional and effective connectivity techniques. Respectively, these tools measure the temporal correlation between remote neurophysiological events (Sporns et al., 2007) and the influence one neural system exerts over another (Friston, 2009). Together, functional and effective connectivity techniques have provided remarkable insight to the brain as a set of interconnected elements embedded within a larger whole.

Increasingly, researchers in the field of developmental cognitive neuroscience are implementing connectivity techniques, making methodological and conceptual strides in the understanding of the developing brain (e.g., Fair et al., 2007, Fair et al., 2008, Dosenbach et al., 2010). These studies have revealed that the complex functional architecture of the brain changes throughout the lifespan. Specifically, functional brain networks in children appear to be composed of multiple decentralized clusters at the local level, while adult function is supported by a more integrated organization distributed throughout the brain (for review, see Vogel et al., 2010). To contribute to this burgeoning literature, the present review summarizes and synthesizes developmental research implementing connectivity techniques to understand the emergence of networks in the brain. In other words, how might mature patterns of connectivity arise as a developmental product of precursors that did not contain these patterns? A dynamic systems framework may provide valuable theoretical principles for conceptualizing the complex interrelations of physical form, time, and process that contribute to the emergence of networks in the human brain.

Dynamic systems theory has been referred to as the broadest and most encompassing of all the developmental theories (Miller, 2002). As defined in the present review, *dynamic systems* is a theoretical approach that describes the behavior of complex networks (Smith and Thelen, 2003). This is different from the more technical use of the term, *dynamical systems*, which refers to a class of mathematical equations that describe time-based systems with particular properties (e.g., Luenberger, 1979). The qualitative principles of this approach are content-independent and have been previously applied to a range of developmental questions such as language acquisition (De Bot et al., 2007), emotion (Lewis and Granic, 2002), and cognition (Thelen, 1996), though have not yet been widely applied to questions of neurobiological development. Under dynamic systems theory, development can only be understood as the multiple, mutual, and continuous interaction of all levels of the developing system. This concept singularly resonates with the growing understanding of the brain as an interconnected system, a series of simpler networks organized into increasingly complex networks, undergoing a changing trajectory throughout the lifespan (Power et al., 2010). The application of this theory to understand the developing brain may help answer such questions as: How can the stable and integrated pattern of the adult neural network emerge from the decentralized patterning typical of a child's brain? How can the local community clusters of a child's brain emerge from a single neuron communicating to another? According to dynamic systems theory, the key to understanding these fundamental developmental questions lies within the process of self-organization. Some form of global order or coordination arises out of the local interactions between the components of an initially disordered system. In other words, development of networks may organically emerge as a product of the system's own activity and the *relationship* between the system's component parts. Connectivity techniques provide a set of tools for researchers to examine *interactions* between elements of the brain. The current review describes tools to assess functional and effective connectivity and describes a framework for understanding large-scale networks. Although the tools described here do not represent the entirety of available techniques implemented to evaluate functional and effective connectivity, they are widely used and have been selected to demonstrate the power of these approaches thematically. Individually and together, these tools have the potential to offer significant contribution in the methodological and conceptual strides being made toward an understanding of the developing brain as a dynamic system. The reader is directed to reviews discussing methods not discussed here, such as Granger causality (Friston et al., 2013).

The general principles of dynamic systems theory may be useful for conceptualizing biological self-organization. The first such principle is the tenet of multicausality, which assumes that the regularities of the mature organism patently emerge from multiple factors, including internal configuration of the system and external changes in the environment that the system responds to. The stable and distributed functional system of the mature brain may be a developmental product of multiple sources, including the system's internal configuration (i.e., intrinsic architecture) and its response to the external environment (i.e., extrinsic architecture). The brain's intrinsic architecture is defined as the spontaneous fluctuations between elements of the neural system in the absence of an explicit task, which can be assessed through the acquisition of functional data such as resting-state. This intrinsic architecture may provide a framework for the moment-to-moment responses that the external world evokes (Fox and Raichle, 2007, Raichle, 2010). Extrinsic networks, defined as in-the-moment coupling of regions in response to external stimuli, may be assessed through task-evoked effective connectivity techniques, such as dynamic causal modeling (DCM). Individually and together, the intrinsic and extrinsic architectures of the brain have the potential to shape the development of functional networks through a shared history of co-activation. Through the use of functional and effective connectivity techniques, researchers can better understand the multiple influences on a developing network, including (1) the stable interactions between elements of the system that are a product of lifetime processes (Buckner et al., 2009) and (2) the more ephemeral, flexible process of one element of the system influencing the dynamics of another element in real-time (Friston, 2009).

Understanding the ways in which these multiple influences interact, not only with each other, but across time, is at the heart of the complexity of developmental science. The second tenet of dynamic systems theory is that development can only be understood as nested processes that unfold over many timescales. For example, neural excitation occurs on the order of a millisecond. Every neural event is the initial condition for the next slice of time. Developmental change occurs over weeks, months, and years. The coherence of time dictates that the dynamics of the smallest timescale (e.g., neural activity) are nested within the dynamics of all other timescales (e.g., developmental growth). Thus, in the study of development, we must be concerned with the interaction of different timescales. Although connectivity techniques have given researchers unprecedented insight to the brain as a complex system, this insight is limited to glimpses of change through discrete windows of developmental time, either on the scale of milliseconds (effective connectivity) or lifetime processes (functional connectivity). Comparisons among snapshots of the brain throughout developmental time may not effectively capture the *process* of change. Given that the intrinsic and extrinsic architectures of the brain shape each other throughout the nested milliseconds and years of developmental time, perhaps a more precise picture of developmental change can be captured through the use of functional and effective connectivity tools in conjunction.

In the current review, the functional examination of intrinsic connectivity is described through resting-state, which is a form of data acquisition during functional magnetic resonance imaging (fMRI). Effective connectivity and subsequent extrinsic architecture is discussed using dynamic causal modeling (DCM) as an example, which is an analytic technique applied to fMRI data. It is worthwhile to mention that psychophysiological interactions (PPI) is a task-based non-directional connectivity technique that has been used widely but is not covered in this review. Graph theory, an analytic technique that can be applied to functional or effective connectivity data, is discussed to provide a framework for understanding large-scale networks. The principles and methodology of each technique will be reviewed, as well as their developmental applications; the limitations and developmental considerations of each approach will be discussed. Finally, current extensions of the technology will be summarized and future directions for the field are proposed. The reader is referred to Power et al. (2010) and Vogel et al. (2010) for excellent and detailed discussions on the use of resting state and graph theory on developmental populations. The purpose of the current review is to use dynamic systems theory as a conceptual framework for the advances being made with functional and effective connectivity techniques; to this end, the studies highlighted are descriptive and not exhaustive.

## 2 Functional connectivity: resting-state

### 2.1 Principles and methodology

Brain regions that often work together form a functional network with a high level of ongoing, strongly correlated spontaneous neuronal activity, without the presence of a task or stimulus (Fox and Raichle, 2007). Resting-state provides a method with which to measure connectivity by examining the level of co-activation between the functional time-series of brain regions during rest (Biswal et al., 1995). These patterns of resting-state correlations are hypothesized to reflect the stable and intrinsic functional architecture of the brain (Buckner et al., 2009).

Biswal and colleagues first demonstrated that ongoing neural activity occurs at rest throughout functionally connected regions of the brain when they revealed a high correlation between the blood oxygen level dependent (BOLD) time-series of the left and right hemispheric regions of the primary motor network in the absence of a task (Biswal et al., 1995). Several studies have since replicated these results, propelling extensive use of the technique in adult (e.g., Fox and Raichle, 2007, Greicius et al., 2003) and developmental populations (e.g., Fair et al., 2007, Fair et al., 2008, Koyama et al., 2011).

The acquisition of resting-state simply involves collecting functional imaging data from participants as they lay in the MRI scanner, fixating on a cross-hair or with eyes closed, while refraining from engaging in any specific cognitive task. One study demonstrated that the BOLD response differed when participants fixated on a cross-hair compared to closing their eyes (Van Dijk et al., 2010), which emphasizes the importance of standardizing data collection techniques given the increasingly widespread use of resting-state.

Once data are collected, one form of analysis to examine the functional connections of a particular brain region is the seed method. Seed-based ROI analyses correlate the resting-state time-series of an a priori brain region of interest against the time-series of all other brain regions, resulting in a functional connectivity map (fcMap; Biswal et al., 1997). The fcMap provides information about which regions the selected seed region is functionally linked to, and to what extent. The simplicity of this analysis affords a strong advantage for seed-dependent methods; however, the information of the fcMap is limited to the functional connections of the selected region, making it difficult to examine connection patterns on a whole-brain scale (Buckner and Vincent, 2007). Additionally, the selection of a priori regions of interest can present a challenge to researchers, given that there are no straightforward prescriptions for how to select seeds.

To examine whole-brain connectivity patterns, methods designed to evaluate general patterns of connectivity have been introduced. There are several model-free methods, though the most widely used are independent component analysis (ICA) and clustering. ICA methods require that the investigator choose the number of components, and then a search is conducted for the existence of spatial sources of resting-state signals that vary together over time and are maximally distinguishable from other sets of signals (Beckmann et al., 2005). Among the advantages of ICA-based methods are their application to whole-brain voxel-wise data and a high consistency of reported results (Damoiseaux et al., 2006). A possible disadvantage of ICA is the complex representation of data that may complicate translation of results to clinical relevance (Fox and Raichle, 2007). Clustering, another model-free method, maximizes the similarity between datapoints by grouping connected points into non-overlapping sub-clusters (Salvador et al., 2005). Although clustering more directly reflects functional connections than ICA, it requires additional seed-like processing steps to compare functional connectivity between patients and healthy volunteers. There are several model-free and seed-based methods available for the analysis of resting-state data, though there is a high degree of overlap and consistency among results of these methods.

**Fig. 1:**
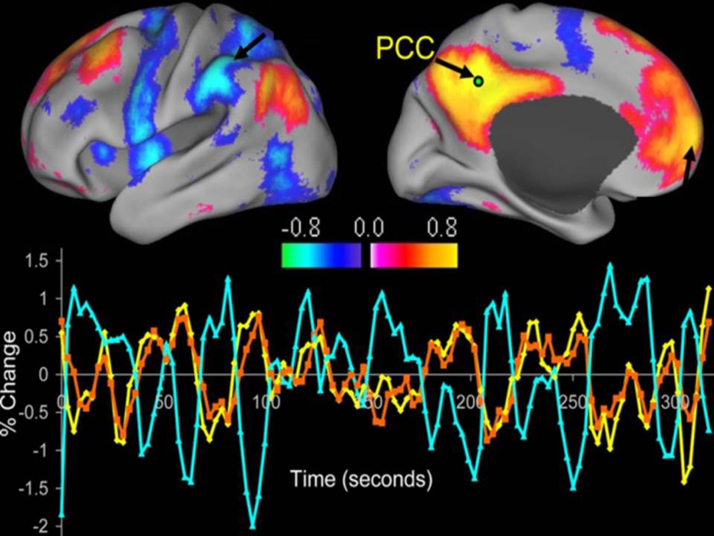
The default mode network. Correlations between a seed region in the posterior cingulate/precuneus (PCC) and all other voxels in the brain during rest, revealing the default mode network. The time course for a single run is shown for the seed region. Regions positively correlated with seed shown in orange, and regions negatively correlated with seed shown in blue. (For interpretation of the references to color in this figure legend, the reader is referred to the web version of the article.)

Adapted from Fox et al., 2005. Copyright © 2005, The National Academy of Sciences.

Since the emergence of resting-state, these methods have been used to identify major functional networks, such as the primary motor, visual, and auditory networks, in addition to higher order cognitive systems (Cordes et al., 2000, Fox and Raichle, 2007, Greicius et al., 2003). Of particular interest is the default mode network (Fig. 1), which consists of the precuneus, medial frontal, and inferior parietal regions (Fox and Raichle, 2007). The regions of this network demonstrate a significantly higher level of neuronal activity during rest, as opposed to when cognitive tasks are performed (Raichle and Snyder, 2007). This suggests that activity of this network is reflecting a default state of neuronal activity.

The use of resting-state in general and the examination of the default mode network in particular can be conducted independent of task performance. This may be advantageous, as differentiating changes in brain activation related to task performance compared to those related to brain maturation has been a challenge in developmental research (Casey et al., 2005). However, it is important to note that even in the absence of an explicit task, the acquisition of resting-state data involves a meta-task in which the subject lies silent and still in the scanner (Poldrack, 2014). This may engage brain regions differentially in children and adults that are unrelated to intrinsic maturation, such as developmental differences in attention.

### 2.2 Developmental applications

Though the technique is still in its infancy, the increasingly widespread application of resting-state has begun to clarify and reveal important principles of functional brain development. The use of resting-state has not only detected the presence of a stable visual network in infants as young as three months old (Kiviniemi et al., 2003), it has revealed that sensorimotor and visual networks undergo differential developmental trajectories, with sensorimotor functional connectivity preceding that in the visual systems (Lin et al., 2008). Another study comparing network connectivity between children, adolescents, and adults found that connectivity of networks associated with social and emotional functions exhibited the greatest developmental effects, while connectivity of networks associated with motor control did not differ between the three groups (Kelly et al., 2009). These studies used resting-state methods with different age groups to confirm the long-hypothesized organizational principle of development, demonstrating that the maturation of motor systems precedes the maturation of systems underlying higher cognition (Chugani et al., 1987). This idea reflects the self-organizing principle of dynamic systems theory, and demonstrates how a system may develop as a hierarchical, non-linear process.

Several resting-state studies have demonstrated that in the development of large-scale brain systems, functional connectivity shifts from a local to distributed architecture. For example, intrahemispheric connectivity within local circuits precedes the development of longer-range interhemispheric connectivity (Fransson et al., 2007, Liu et al., 2008). Others have found that nodes within the default mode network are sparsely connected in children and strongly functionally connected in adults (Fair et al., 2008). One group used 5 minute-long resting-state scans from a sample of typically-developing subjects across a range of ages to make accurate predictions about individuals’ brain maturity across development (Dosenbach et al., 2010). Results indicated the greatest contributor to predicting individual brain maturity was the strengthening of the adult brain's major functional networks, as well as the sharpening of the boundaries between these networks (Dosenbach et al., 2010).

### 2.3 Limitations

In the study described above (Dosenbach et al., 2010), the group later discovered that despite meticulous attention to exclusion of high-motion subjects, substantial changes in the timecourses of resting-state data appeared to have been introduced by relatively minor subject movements in the scanner (Power et al., 2012). This has been a concern that has since received much attention in the developmental literature, as motion artifact specifically tends to enhance short-range connectivity and diminish long-distance connectivity among network nodes (Power et al., 2012). This has particular implications for conclusions drawn from developmental resting-state studies because it is unclear to what extent the finding that networks undergo a local to long-range trajectory has been influenced by greater motion in children compared to adults. Given the potentially large implications the relatively small movements in the scanner may have, it is of critical importance to understand how to best model and account for subtle motion (Power et al., 2012; Satterthwaite et al., 2012, Van Dijk et al., 2012). For a description of how different preprocessing strategies may alter the way motion artifact manifests, the reader is referred to Satterthwaite et al. (2013).

Additionally, raw resting-state data is influenced by physiological noise (Biswal et al., 1995). Researchers have worked to develop techniques to remove this noise, such as spatial smoothing (to improve signal-to-noise ratio), temporal filtering (to remove signal contributed by physiological sources), and whole-brain signal regression (to account for noise sources such as motion) (Supekar et al., 2009), although the development of these preprocessing techniques has introduced new concerns about the interpretation of processed data (Cole et al., 2010).

Resting-state is still a novel method, and acquisition and analysis techniques undergo continual standardization and refinement. Although the conclusions drawn from resting-state data appear to be relatively robust, only time will tell the extent of potentially spurious findings due to motion as researchers continue to search for the most effective preprocessing for raw resting-state data. The technique, though still evolving, provides invaluable information on the intrinsic functional architecture of the developing brain.

## 3 Effective connectivity: dynamic causal modeling (DCM)

### 3.1 Principles and methodology

Advancements in the area of effective connectivity allow for an examination of co-activation between neural regions during performance of a task or experimental manipulation (Friston et al., 2003); specifically, dynamic causal modeling (DCM) provides a statistical tool to infer causal architecture of coupled or distributed systems. In other words, the effect of one neural region influencing another given an external stimulus can be captured in real-time (Friston, 2009, Stephan et al., 2010). Thus, the relationships between elements of the system can be examined in a directional, task-specific and extrinsic manner.

DCM may be used with data collected during functional magnetic resonance imaging (fMRI) to create a simple model of neural dynamics in a network of *n* interacting neural regions (Friston et al., 2003). This dynamic model estimates how changes in neuronal activity in one node are caused by activity in another. Since its inception (Friston et al., 2003), a number of developments have improved and extended DCM as a tool to furnish an explicit generative model of how observed data are caused (Friston, 2009). This means that the exact form of the DCM changes with each application and speaks to its progressive refinement.

**Fig. 2:**
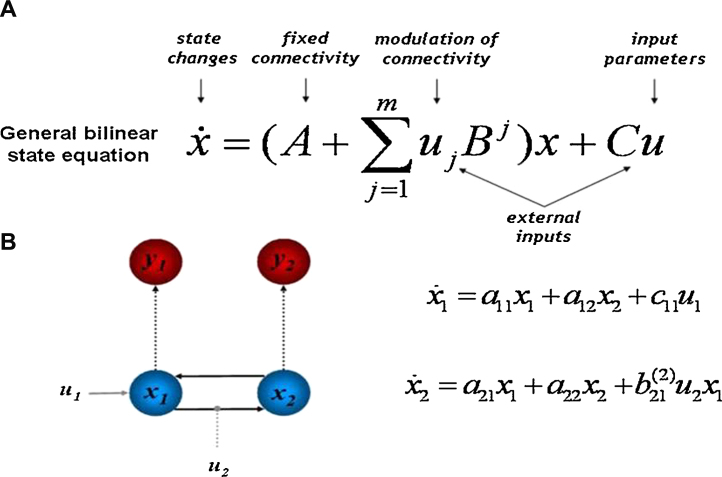
Dynamic causal modeling (DCM). The bilinear state equation for DCM with fMRI (a). An example of a DCM consisting of two nodes (*x*_1_, *x*_2_) shown in blue. Black arrows represent functional connections. Gray arrows represent exogenous inputs (*u*_1_, *u*_2_). Dotted arrows represent changes from hidden neural states to measurable hemodynamic observations, shown in red. (For interpretation of the references to color in this figure legend, the reader is referred to the web version of the article.)

Adapted from Stephan et al. (2007). Reprinted with kind permission from Springer Science and Business Media: [Journal of Biosciences] copyright (2007).

Dynamic models are created through the use of a general bilinear state equation resulting from a Taylor approximation of how changes in neural activity in one node *x*_1_ are caused by activity in another node *x*_2_ (Fig. 2a, from Stephan et al., 2007). Within this equation, the A-Matrix refers to the coupling between regions in the absence of experimental manipulation (called fixed or average connectivity) and the B-Matrix refers to changes in coupling strength caused by the experimental manipulations. The B-matrix (*u*_*j*_) embodies influences of experimental manipulations that cause perturbations of neural states in these nodes. The inputs to the model are denoted by *u* and the C-Matrix indexes the direct influences on an area (Fig. 2b, from Stephan et al., 2007). DCMs are created and analyzed with configurations of forward and backward self-connections and their modulations, and Bayesian model comparison is implemented to select the model that best fits the data. In addition, DCM provides a parameter estimate of the strength of the connection and the strength of the modulation by an experimental condition. In this way, DCM offers a powerful statistical tool that evaluates reciprocal and hierarchical functional organization within a particular dataset in response to extrinsic task manipulations.

### 3.2 Developmental application

Beyond the ability to evaluate functional communication between nodes in a causal and directional manner, one of the strengths of DCM is the capacity to incorporate exogenous inputs in the analyses. In this way, the effects of task manipulations on hidden neuronal states and their interactions are able to be assessed and quantified (Stephan et al., 2010). These analyses allow researchers to investigate functional organization and connectivity in an in-the-moment manner, examining how communication between brain regions changes as a result of experimental manipulations, context, or time. Despite its potential utility in characterizing complex, context-dependent developmental processes, relatively few studies have implemented DCM developmentally. One study has implemented DCM to examine how three components of reward neurocircuitry, the nucleus accumbens, thalamus, and anterior insula, function as a network during gain or loss anticipation in adults and youth (Cho et al., 2013). Both groups demonstrated a broader set of significant connections found for the loss condition than the gain condition, reflecting context-dependent findings, though no statistically significant between-group comparisons were found, suggesting that adults and adolescents use this incentive-processing network in a similar manner (Cho et al., 2013). Another recent study implemented DCM to examine the effective connectivity underlying increases in relational integration during relational reasoning across development (Bazargani et al., 2014). Findings demonstrated distinct developmental effects on the strength of long-range versus short-range connections, and the modulatory connections of relational integration increased with age, providing converging evidence from multiple connectivity techniques regarding the segregation and integration that are proposed to occur during development (Vogel et al., 2010). There is also evidence that effective coupling is associated with brain maturation, such that top-down modulatory effects in adolescents differed from those in adults, suggesting their slow formation in human ontogenesis (Fornari et al., 2014). Although there are relatively few studies using DCM in developmental populations, more recent studies are beginning to emerge, and the use of this statistical tool appears to be promising for understanding causal neurodevelopmental processes.

### 3.3 Limitations

It is important to note that DCM is not an exploratory tool, as its implementation requires the formulation of a priori hypotheses and the pre-specification of a set of models for testing. However, a method has been developed to explore very large numbers of models using a post hoc procedure in which only the full model is inverted, and the model evidence for any reduced model is obtained using a search procedure. This search procedure takes a subset of parameters with the least amount of evidence and searches over all reduced models within that subset (Friston et al., 2011). Additionally, DCM is dependent upon experimental manipulations; in the past, DCM was not suitable for use with resting-state data, although recent advancements allow for the application of DCM analyses on task-free resting-state data (i.e., stochastic DCM) and in exploratory settings (Friston et al., 2011). It is evident that, as with any new technology, the use of DCM relies upon frequent refinements as new knowledge is continually gained as to the accuracy of the technique. A number of developments have improved and extended DCM since its inception (Friston et al., 2003). For fMRI, models of precise temporal sampling (Kiebel et al., 2007), multiple hidden states per region (Marreiros et al., 2008), a refined hemodynamic model (Stephan et al., 2007), and a nonlinear model (Stephan et al., 2008) have been introduced. In other words, the analytic technique has been tailored for use with fMRI through the incorporation of methodological refinements related to timing and hemodynamic response in the model, which was not originally developed for use with fMRI.

## 4 The brain as a large, complex network: graph theory

### 4.1 Principles and methodology

In addition to the formation of multiple subnetworks, the brain forms one integrative network that links all brain regions into a single, complex system. Graph theory provides a theoretical framework to examine complex systems, and can reveal important information about the local and global organization of functional brain networks (Bullmore and Sporns, 2009).

**Fig. 3:**
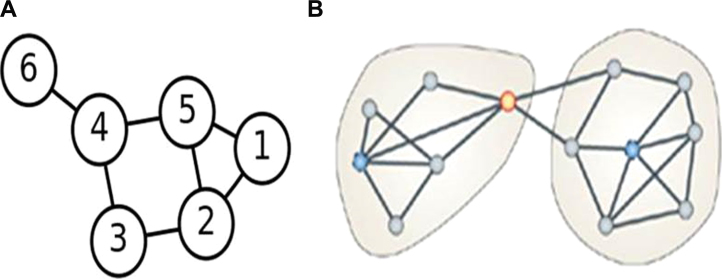
Illustration of a graph. Graphs are made up of *nodes* and lines called *edges* that connect them (a). With the use of graph theory, researchers have characterized the small-world architecture of neural networks. Structurally and functionally related regions are densely connected within hubs, or modules; these modules are sparsely connected with each other (b).

Adapted from Bullmore and Sporns (2009). Reprinted by permission from Macmillan Publishers Ltd: [Nature Reviews Neuroscience] copyright (2009).

Graph theory can be used to model many types of dynamic processes and relationships in physical, biological, social, and information systems (Bondy and Murty, 1976). The application of graph theory has aided in the examination of such varied grids as aircraft flight patterns, biological systems, and the internet, since complex networks tend to consist of similar topological patterns between their constituent parts. With respect to the brain, functional networks can be described as graphs that are composed of nodes (i.e., brain regions) that are linked by edges, representing functional connectivity (i.e., correlations between timeseries) (Fig. 3a, from Bullmore and Sporns, 2009). Thus, within the graph theory framework, the nodes of the brain network are represented as regions, which may be based on a predefined anatomical region of interest or fMRI voxels. The level of connectivity between two regions is computed as the level of correlation between the time-series of the two brain regions. Computing the level of functional connectivity between all possible pairs of nodes and determining the existence of a functional connection by using a predefined statistical threshold results in a graph representation of the functional brain network. This graph representation allows for the examination of network organization using graph theory.

A network's topological patterns are evaluated using the key properties of graph theory: clustering coefficient, characteristic path length, node degree, centrality, and modularity (Sporns et al., 2004). The *clustering coefficient* of a graph provides information about the level of local clustering within a graph, expressing how well the neighbors of a node are connected amongst themselves. This provides a measure of how much spatially-closer brain regions are connected with each other, or local connectedness of the network. The level of global connectivity of the network can be assessed with the *characteristic path length* of a graph, which describes how close, on average, a node of the network is connected to every other node in the network. This provides information on how efficiently information can be integrated between different systems. The *degree* of a node describes the number of connections of a node and provides information about the existence of highly connected hub nodes in the brain network. Additionally, *centrality measures* indicate how many of the shortest travel routes within a network pass through a specific node, providing further information on the formation of hubs within networks. If a node has a high level of centrality, it facilitates a large number of shortest routes in the network, indicating that it has a key role in the overall communication efficiency of a network. Finally, the level of *modularity* of a network describes the extent that groups of nodes in the graph are connected to other members of their own group, establishing sub-networks within the greater network. Taken together, these values of graph theory provide important information about the structure of a network and may characterize a specific organization style (e.g., small-world, modular) of that network.

Neuroimaging research applying graph theory on resting-state data has revealed small-world architecture of functional brain networks across development (Achard et al., 2006, Sporns et al., 2004, Bassett and Bullmore, 2006; Power et al., 2012). In other words, most nodes are not direct neighbors, but most nodes can be reached from every other by a small number of steps. Studies reveal highly clustered large-scale cortical networks, with most existing pathways linking areas that are spatially close and functionally related (Power et al., 2010). These clusters, or modules, are then connected by specialized hub regions. The long-range connections between different modules, though few in number, keep the overall path lengths across the network low (Fig. 3b, from Bullmore and Sporns, 2009).

Small-world network organization is an efficient organization for flow of information (Bullmore and Sporns, 2012). While both young and older adults exhibit small-world network organization, the topological roles of the specific brain regions as well as the inter-regional connectivity appear to differ significantly between the two groups (Power et al., 2010). Converging evidence from multiple studies suggests that whereas children demonstrate similar small-world architecture as adults (Fair et al., 2009, Supekar et al., 2009), the organization of individual sub-networks has a protracted time course. Developmental studies implementing graph theory have the potential to reveal the features of large-scale network development within the brain (Kashtan and Alon, 2005).

### 4.2 Developmental applications

The ontogeny of the large-scale functional organization of the brain is still not well understood, though studies have begun to use network analyses to reveal that children and adults display similar small-world architecture at a global level to maximize efficiency, with specific group differences in hierarchical organization and interregional connectivity (Fair et al., 2009, Supekar et al., 2009). For instance, while adult networks consist of prominent cortico-cortical connections, children tend to have stronger and more abundant connections between subcortical and cortical regions (Supekar et al., 2009). Specifically, subcortical areas appear to be more strongly connected with primary sensory, association, and paralimbic areas in children, whereas adults show stronger cortico-cortical connectivity between paralimbic, limbic, and association areas. The small-world nature of the brain may allow for the formation of neural networks that work together in a highly stable yet flexible manner, perhaps to allow for adaptive change based on experience and environment.

Additionally, more mature brains exhibit greater hierarchical organization, with more regions involved in longer-distance clusters of activity. Networks with greater hierarchy are characterized by high degree nodes, which exhibit low clustering. Hierarchical networks contain small densely connected clusters that combine to form large, less interconnected clusters, which further combine to form larger and lesser interconnected clusters (Ravasz and Barabási, 2003). Hierarchical networks form to support top-down relationships between nodes while minimizing wiring costs (Ravasz and Barabási, 2003). Lower levels of hierarchical organization in children may allow for more flexibility in network growth on the basis of experience.

Finally, the development of large-scale neural networks is characterized by a weakening of short-range functional connectivity and a strengthening of long-range functional connectivity. Functional connectivity between more proximal anatomical regions is significantly higher in children, whereas functional connectivity between more distal anatomical regions is significantly higher in adults (Supekar et al., 2009). This suggests a pattern of higher short-range functional segregation in children and higher long-range functional integration in adults, contributing evidence for the hypothesis that the development of large-scale neural networks involves a dual process of functional segregation and integration (Fair et al., 2007). In the first large-scale study to examine the lifespan trajectories of brain network properties based on graph theory, a recent study (Cao et al., 2014) revealed linear decreases in modularity and inverted U-shaped trajectories of local efficiency. Together, these findings provide insights into the development of large-scale brain organization with the use of networks analysis. In this way, graph theory can provide a powerful statistical framework to characterize the development of brain systems in a comprehensive manner, considering not only relationships within a given system, but also how these relationships are situated within wider network contexts (Power et al., 2010).

### 4.3 Limitations

Such emerging findings use graph theory to provide important insight into the development of functional neural systems; however, limitations must be addressed. The functional network of the brain is comprised of neurons and columns, physical elements that have a limited ability of being accurately measured and defined in humans. For graph theory analyses, inferences must be made when nodes are defined as voxels, or anatomically- or functionally-defined regions of interest. Definitions of nodes are particularly difficult in developmental research, as the nodes are likely not the same across the sample. The use of graph theory requires careful selection of nodes and an understanding that obtained graphs are only as accurate as the nodes. Given that it is impossible to fully know the true definition of functional nodes in the human brain, graphs likely contain some amount of distortion (Power et al., 2010).

## 5 Conclusions and future directions

The implementation of graph theory to resting-state data has demonstrated that the brain is a remarkable and complex hierarchy of multiple subsystems within one dynamic system. It is important to note that graph theory may also be applied to effective connectivity data, and as an increasing number of developmental studies begin to use DCM, a directional understanding of the influence of functional regions within the context of the larger system may be possible (Bullmore and Sporns, 2009). The growing understanding of the brain as a developing, hierarchical network that has been attained through the implementation of functional and effective connectivity techniques underscores the potential utility of conceptualizing these findings within the framework of dynamic systems theory. The brain has been demonstrated to be a dynamic system, a set of interconnected elements embedded within a larger whole.

The development of the brain is influenced by multiple elements, with no one element taking priority. This means that no single element (e.g., one neuron, neural region, or neural system) drives development; rather, it is the interaction between elements that is important. The use of functional connectivity techniques such as resting-state captures the intrinsic and stable functional networks of the brain, while effective connectivity assesses the moment-to-moment influence of one element of the system over another. In the same way that a dynamic system may generate novelty through interactions between individual elements of the system, the combined implementation and interpretation of connectivity techniques may have the potential to reveal new insight that has not yet been attained through use of these tools individually. Specifically, examining functional and effective connectivity data in conjunction may capture the process of change and the emergence of novel patterns that may arise from activity within the system itself (Smith and Thelen, 2003). We speculate that the use of connectivity tools in conjunction may allow developmental researchers to gain a deeper understanding of the multiple and reciprocal interactions that occur across multiple hierarchies and timescales of the system.

Previous examinations of coupling between the extrinsic and intrinsic architecture of the brain has been done through the use of large-scale meta-analytic approaches (Toro et al., 2008, Smith et al., 2009). While these large-scale approaches are powerful, they do not capture the moment-to-moment covariation of BOLD response across trials (Mennes et al., 2013). As an alternative, a recent study investigated regional variation at a participant level by computing the spatial correlation between patterns of intrinsic functional connectivity acquired through resting-state and patterns of task-evoked functional connectivity for each voxel in the brain (Mennes et al., 2013). While this study was done in adults, examining the coupling between these two architectures has the potential to capture the interaction between two systems that operate within distinct developmental time. The intrinsic architecture of the brain, a relatively stable system, may undergo a slow trajectory of protracted change over the lifetime. Extrinsic architecture reflects co-activation of neural regions to an external response in the slice of time in which it occurs. By examining the coupling of these architectures across the lifespan, new insight may be gained into the organic emergence of new patterns through the interaction of lifetime processes and in-the-moment change.

## Conflict of interest

There are no conflicts of interest to report.
